# Technics

**Published:** 1887-06

**Authors:** 


Technics sends bi-weekly a newsy sheet to our sanctum. It contains interesting matter, of course entirely from the old school side, except when it occasionally gives notice of a wonderful discovery of some new medicine—long ago used by the homœopathic school.

